# Risk factors for severe hearing loss in Susac syndrome: A national cohort study

**DOI:** 10.1111/ene.16211

**Published:** 2024-01-18

**Authors:** Marion Peyre, Arthur Mageau, Marie‐Cécile Henry Feugeas, Serge Doan, Caroline Halimi, Isabelle Klein, Tiphaine Goulenok, Chrystelle François, Marie‐Paule Chauveheid, Thomas Papo, Karim Sacré, the French Susac Study Group, Jean‐Francois Alexandra, Olivier Aumaitre, Naima Beldjoudi, Marie Bodenant, Jean Capron, Thomas de Brouker, Elisabeth Medeiros de Bustos, Sabrina Debruxelles, Nicole Delory, Antoine Dossier, Emmanuel Ellie, Fatima Farhi, Sophie Godard‐Ducceschi, Bertrand Godeau, Deborah Grosset‐Janin, Mohamed Hamidou, Cedric Laouenan, Bertand Lapergue, Alain Le Quellec, Shirine Mohamed, Luc Mouthon, Jean‐Baptiste Noury, Diane Rouzaud, Guillaume Turc, Anne Wacongne

**Affiliations:** ^1^ Department of Internal Medecine, Hospital Bichat‐Claude Bernard, Assistance Publique Hôpitaux de Paris Université Paris Cité Paris France; ^2^ Department of Radiology, Hospital Bichat‐Claude Bernard, Assistance Publique Hôpitaux de Paris Université Paris Cité Paris France; ^3^ Department of Ophthalmology, Hospital Bichat‐Claude Bernard, Assistance Publique Hôpitaux de Paris Université Paris Cité Paris France; ^4^ Department of Otolaryngology ‐ Head and Neck Surgery, Hospital Bichat‐Claude Bernard, Assistance Publique Hôpitaux de Paris Université Paris Cité Paris France; ^5^ Department of Radiology Clinique Alleray‐Labrouste Paris France; ^6^ Centre de Recherche sur l'Inflammation, INSERM UMR1149, CNRS ERL8252, Faculté de Médecine site Bichat Laboratoire d'Excellence Inflamex Paris France

**Keywords:** hearing loss, risk factors, Susac syndrome

## Abstract

**Background:**

Nonreversible hearing loss (HL) is the main sequelae of Susac syndrome (SuS). We aimed to identify risk factors for HL in SuS.

**Methods:**

The CARESS study is a prospective national cohort study that started in December 2011, including all consecutive patients with SuS referred to the French reference center. The CARESS study was designed with a follow‐up including fundoscopy, audiometry, and brain magnetic resonance imaging at 1, 3, 6, and 12 months after diagnosis and then annually for 5 years. The primary outcome was the occurrence at last follow‐up of severe HL defined as the loss of 70 dB in at least one ear on audiometry or the need for hearing aids.

**Results:**

Thirty‐six patients (female 66.7%, median age 37.5 [range 24.5–42.5] years) included in the clinical study were analyzed for the primary outcome. Thirty‐three patients (91.7%) had cochleovestibular involvement at SuS diagnosis including HL >20 dB in at least one ear in 25 cases. At diagnosis, 32 (88.9%), 11 (30.6%), and 7 (19.4%) patients had received steroids, intravenous immunoglobulin, and/or immunosuppressive (IS) drugs, respectively. After a median follow‐up of 51.8 [range 29.2–77.6] months, 19 patients (52.8%) experienced severe HL that occurred a median of 13 [range 1.5–29.5] months after diagnosis. Multivariable analysis showed that the odds of severe HL were lower in patients who received IS drugs at diagnosis (OR 0.15, 95% CI 0.01–1.07, *p* = 0.058).

**Conclusions:**

Severe HL in SuS is associated with the absence of IS drugs given at diagnosis. Our findings support the systematic use of IS drugs in SuS.

## INTRODUCTION

Susac syndrome (SuS) is a rare disease affecting mainly young women and characterized by an occlusive microvessel disease limited to the brain, retina, and inner ear [1, 2, 3], although spinal cord and skin involvement have been reported [4]. The pathophysiology is not fully elucidated [5] but recent advances support the hypothesis that SuS is an autoimmune endotheliopathy affecting the cerebral, retinal, and cochlear small vessels [6]. SuS is recognized in the presence of the pathognomonic triad associating: (i) subacute encephalopathy with corpus callosum lesions on brain magnetic resonance imaging (MRI); (ii) eye involvement with occlusions of the branches of the central artery of the retina; and (iii) cochleovestibular damage with hearing loss (HL) predominating at low frequencies on audiometry [7]. Nonreversible HL is the main long‐term damage of SuS found in about 50% of patients [8, 9] and might require hearing aids [10, 11]. There are no known predictive factors for poor outcomes.

We herein leverage the prospective national cohort study (CARESS study) to identify risk factors for severe HL in SuS.

## METHODS

### Study population

All patients participated in the national multicenter CARESS (Phenotypic and Etiological Characterization of Susac Syndrome ‐ National Clinical Research Hospital Program) study. The CARESS study is an ongoing cohort study that started in December 2011 and included all consecutive case of patients with SuS referred to the French reference center (Department of Internal Medicine, Bichat Claude Bernard Hospital, Paris). Inclusion criteria were a minimal age of 18 years and SuS defined either by (i) the triad of encephalopathy with typical brain MRI abnormalities, cochleovestibular damage including unilateral or bilateral sensorineural HL on the audiometry and multiple occlusions of retinal central artery branches, and/or retinal vasculitis on retinal fluorescein angiography or (ii) at least two of the three aforementioned criteria without any alternative diagnosis, as required by current diagnostic criteria [7].

### Data collection

Age at diagnosis, gender, physical examination, fundoscopy, retinal angiography, visual acuity, visual field, audiometry, cerebrospinal fluid (CSF), brain MRI, and treatment data were systematically collected. The CARESS study was designed with a follow‐up including fundoscopy, audiometry, and brain MRI at 1, 3, 6, and 12 months after diagnosis and then annually for 5 years and/or in the case of a relapse. A relapse was defined by new clinical symptoms or signs AND new abnormalities on retinal angiography, audiometry, or brain MRI leading to treatment intensification. Most MRI scans were obtained with a 3T MRI scanner performed at Bichat Hospital (GE Medical Systems Discovery MR750). All brain MRIs were analyzed by an experienced neuroradiologist (M.‐C.H.F.) that used our institutional Picture Archiving and Communication System (PACS, Carestream Health Inc., USA) and was blinded to clinical data.

### Primary outcome

The primary outcome was the occurrence of severe HL at last follow‐up. Severe HL was defined as the loss of 70 dB in at least one ear on audiometry or the need for hearing aids.

### Standard protocol approvals, registrations, and patient consents

The CARESS study has been approved by the Committee for the Protection of Persons (CPP, Ile de France 1, Paris, France; IRB00008522). All patients signed informed consent. Detailed inclusion and exclusion criteria for the CARESS study are available on the ClinicalTrials.gov portal (Identifier: NCT01481662).

### Statistical analysis

Continuous variables are expressed as median [1st quartile (Q1)–3rd quartile (Q3)]. Categorical variables are expressed as frequencies and percentages. Data were compared using chi‐square test (or Fisher test) for dichotomous variables and Mann–Whitney test for continuous variables. To determine which characteristics were associated with the primary outcome, we performed univariate and multivariable logistic regression procedures calculating odds ratios (ORs) and their 95% confidence intervals (95% CIs) using the Firth's penalized method for small samples [12]. Clinically relevant variables associated with the primary outcome in univariate analysis with a *p*‐value < 0.05 were considered for the multivariable analysis. Statistical analysis was performed using R® software (Version 1.0.153). A *p‐*value < 0.05 was considered statistically significant.

Trial Registration CARESS study ClinicalTrials.gov portal Identifier: NCT01481662 https://clinicaltrials.gov/ct2/show/NCT01481662?term=caress&draw=2&rank=5.

## RESULTS

### Characteristics of patients

Fifty‐seven patients from 20 different centers in France with SuS were included in the CARESS (ClinicalTrials.gov Identifier NCT01481662) study. Among them, 21 patients were excluded from the analysis because of missing audiometry (*n* = 20) or cophosis prior to SuS (*n* = 1). Overall, 36 SuS patients were analyzed for the primary outcome (Figure S1, flowchart). Twenty‐four patients (66.7%) were women and the median age at diagnosis was 37.5 [range 24.5–42.5] years. The triad was complete in 31 (86.1%) patients at disease onset, and 3 (8.3%) additional patients completed the triad during disease course. At diagnosis, all but 4 patients (88.9%) received steroids including high‐dose intravenous pulses in 93.8% (*n* = 30/32) of cases. Steroids were associated with intravenous immunoglobulin (IVIG) therapy (*n* = 11) and/or immunosuppressive (IS) drugs (cyclophosphamide *n* = 5, azathioprine *n* = 1, or methotrexate *n* = 1). Antiplatelet therapy was given in all but two patients (94.4%). Twenty‐eight (77.8%) patients relapsed at least once during follow‐up. All but one relapses occurred within the 12 months (median 2 [range 1–5.25] months) after disease onset with a median number of 1 [range 1–2] relapses per patient (Table 1).

**TABLE 1:** Characteristics of Susac syndrome patients (*N* = 36).

Characteristic	Value
Age, years	37 [25–43]
Diagnostic delay, months	2 [1–4]
Women	24 (66.7)
Triad at onset	31 (86.1)
Neurologic signs	36 (100)
Headache	28 (77.8)
Encephalopathy	19 (33.3)
Behavioral, conduct, or mood disorder	18 (50)
Motor impairment	12 (33.3)
Ophthalmic signs	33 (91.7)
Visual field loss	8 (22.2)
Visual acuity loss	8 (22.2)
Arterial occlusion	30 (83.3)
Hyperfluorescence	11 (30.5)
Cochleovestibular signs	33 (91.7)
Tinnitus	16 (44.4)
Dizziness	15 (41.7)
Ataxia	7 (19.4)
Hearing loss, >20 dB	25 (69.4)
Hearing loss, >40 dB	21 (58.3)
Cerebrospinal fluid	
Proteins >0.4 g/L	30 (83.3)
Proteins, g/L	1.2 [0.9–1.8]
Brain MRI^a^	
Number of DWI‐HL	40 [12–131]
Treatment at diagnosis	
Corticosteroid	32 (88.9)
High dose pulse	30 (83.3)
IS drugs	7 (19.4)
IVIG	11 (30.6)
Antiplatelet therapy	34 (94.4)
Follow‐up	
Duration, months	51.8 [29.2–77.6]
Relapses per patient	1 [1–2]

*Note*: Quantitative variables are expressed as median [1st quartile–3rd quartile]. Qualitative variables are expressed as number (percentage). Immunosuppressive drugs included cyclophosphamide (*n* = 5) methotrexate (*n* = 1), and azathioprine (*n* = 1).

Abbreviations: dB, decibel; DWI‐HL, hyperintense lesions on diffusion‐weighted imaging; IS, immunosuppressive; IVIG, intravenous immunoglobulin; MRI, magnetic resonance imaging.

^a^ On first MRI available (*n* = 24) performed during the 3 months following the onset of symptoms in all but one patient.

### Cochleovestibular involvement at diagnosis

**FIGURE 1:**
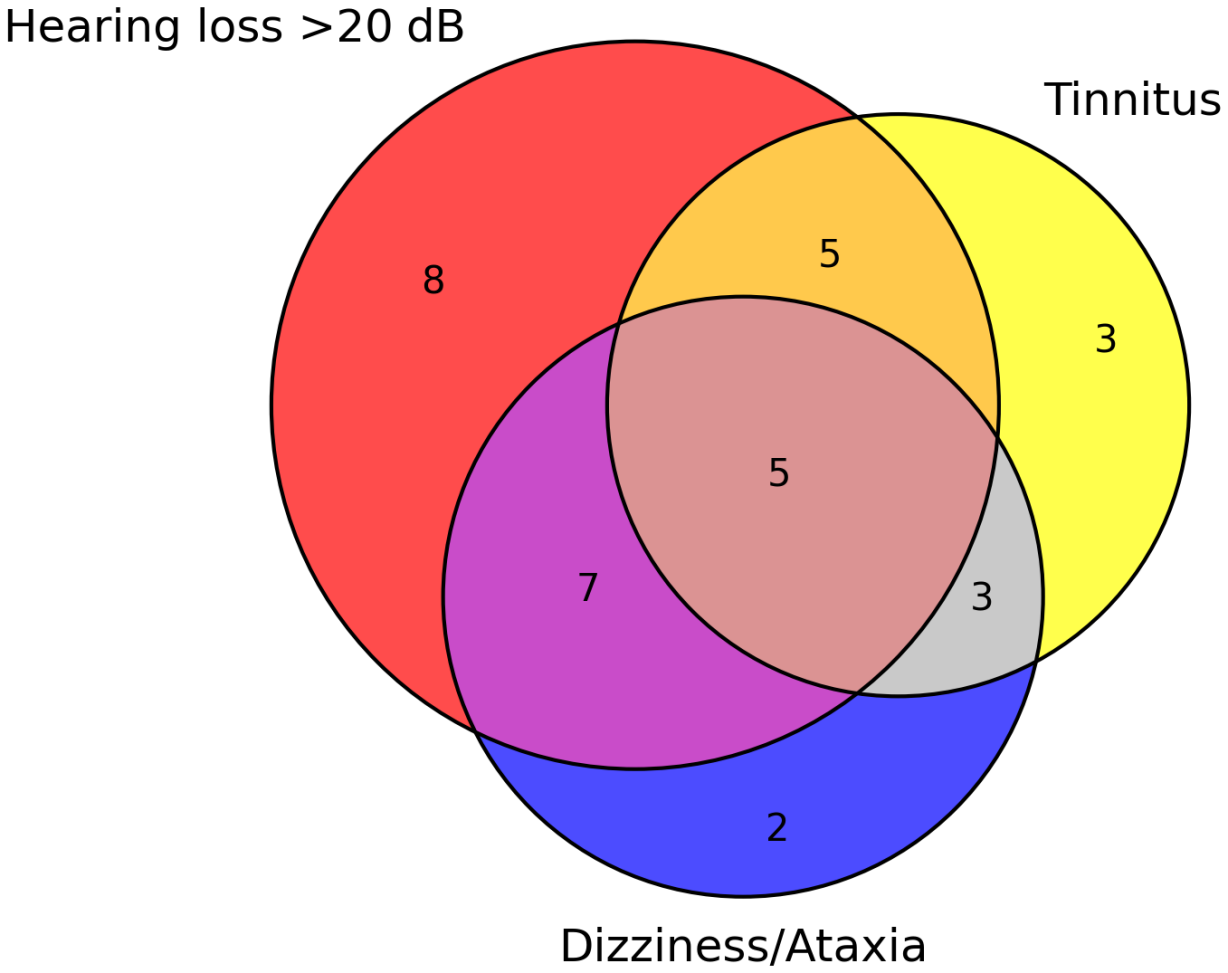
Cochleovestibular involvement at Susac syndrome (SuS) diagnosis. Venn diagram illustrating the relationships between hearing loss (red), tinnitus (yellow), and dizziness/ataxia (blue) in SuS patients with cochleovestibular involvement at diagnosis (*n* = 33).

All but three patients (*n* = 33, 91.7%) had cochleovestibular involvement at SuS diagnosis including tinnitus (*n* = 16), dizziness (*n* = 15), ataxia (*n* = 7), and HL >20 dB in at least one ear (*n* = 25). Most patients (*n* = 20, 57.1%) complained of multiple cochleovestibular signs but HL was isolated in eight cases (24.2%) (Figure 1). Median HL was of 50 [range 40–70] dB, predominately in the low to midtone range frequencies in 16 (64%) patients and was bilateral in 11 (44%) patients. Of note, seven patients did not complain from HL at diagnosis despite an audiometry showing an actual HL ranging from 20 to 70 dB (median 40 [range 32.5–42.5] dB).

### Risk factors associated with severe hearing loss

**FIGURE 2:**
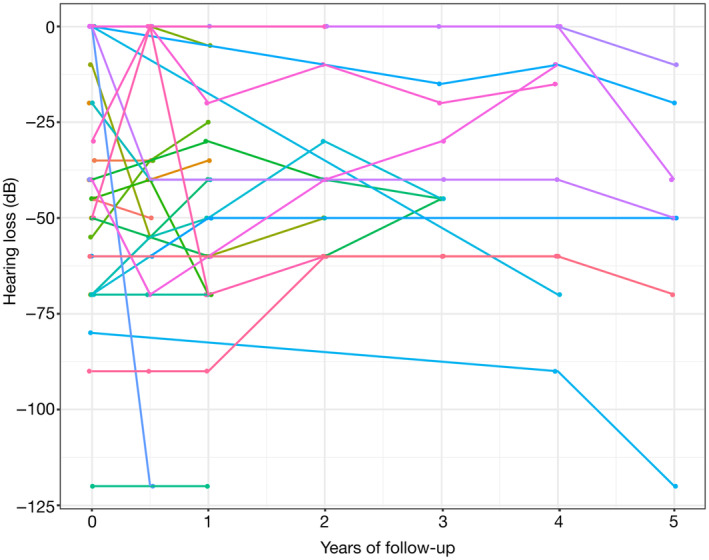
Audiometric findings at disease onset and during follow‐up. Spaghetti plot showing longitudinal audiometry decibels (dB) in Susac syndrome patients from disease onset to last follow‐up. The mean hearing loss in at least one ear was of 9.7 dB (SD 34.9 dB) over time (–39 dB [SD 32 dB] at disease onset versus –49 dB [SD 25 dB] at last follow‐up (Wilcoxon test, *p* = 0.16). The connecting lines between measurements are matched at the individual level. SD, standard deviation.

**FIGURE 3:**
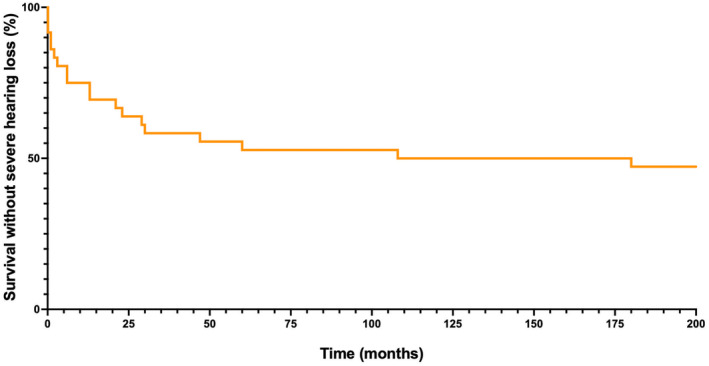
Severe hearing loss during follow‐up. The time delay to reach the primary outcome in Susac syndrome (SuS) patients with severe hearing loss at last follow‐up (*n* = 19) is shown. The number of patients is represented according to the elapsed time since the first audiometry. During follow‐up, patients performed 3 [range 2–3.25] audiometries in the year following SuS diagnosis and 7 [range 5–9] over time. The median time to reach the primary outcome was 13 [1.5–29.5] months.

The primary outcome was evaluated after a median follow‐up of 51.8 [range 29.2–77.6] months while SuS was in remission for more than 12 months in all but one patient with no later relapse. Although HL slightly improved in 11 patients (median increase of 22.5 [range 10–31.25] dB in a least one ear) during follow‐up, 19 patients (52.8%) experienced severe HL that occurred at onset (*n* = 9) or at relapse (*n* = 10) (Figures 2 and 3). Severe HL was more frequent in women, in patients who experienced visual loss, in patients who did not experience encephalopathy and in those who did not receive IS drugs at SuS diagnosis (Table 2). Brain lesion load at diagnosis, evaluated through DWI, was not associated with severe HL. Among the 11 patients who had no HL at SuS diagnosis, 6 experienced HL during follow‐up. None of the six patients had received IS drugs at SuS diagnosis while two of the five patients who did not experience severe HL had received IS treatment. Eventually, multivariable logistic regression analysis, involving gender, encephalopathy, and IS drugs as predictor variables, showed that odds of severe HL were lower in patients who had received IS drugs at SuS diagnosis (OR 0.15 [range 0.01–1.07], *p* = 0.058) (Table 2).

**TABLE 2:** Risk factors associated with severe hearing loss in Susac syndrome patients.

	Severe HL (*n* = 19)	No severe HL (*n* = 17)	OR (95% CI)^a^	*P*‐value	OR (95% CI)^b^	*P*‐value
Age, years	39 [25–46]	37 [25–40]	1.02 [0.96–1.08]	0.61		
Women	16 (84.2)	8 (47.1)	5.57 [1.28–6.14]	0.02	4.66 [0.87–29.2]	0.73
Triad at onset	17 (89.47)	14 (82.35)	1.69 [0.29–11.4]	0.56		
Neurologic signs	19 (100)	17 (100)	–			
Headache	14 (73.68)	14 (82.35)	0.64 [0.13–2.87]	0.56		
Encephalopathy	6 (31.58)	13 (76.47)	0.16 [0.04–0.62]	0.01	0.43 [0.08–2.30]	0.31
Behavioral, conduct, or mood disorder	11 (57.89)	7 (41.18)	1.89 [0.53–7.12]	0.33		
Motor impairment	6 (31.58)	6 (35.29)	0.85 [0.22–3.30]	0.81		
Ophthalmic signs	17 (89.47)	16 (94.12)	1.13 [0.16–8.15]	0.90		
Visual field loss	3 (15.79)	5 (29.41)	0.48 [0.10–2.18]	0.34		
Visual acuity loss	8 (42.11)	0 (0)	25.9 [2.75–3467]	<0.01		
Arterial occlusion	14 (73.68)	16 (94.12)	0.24 [0.02–1.40]	0.12		
Hyperfluorescence	4 (23.53)	7 (41.18)	0.36 [0.08–1.53]	0.17		
Cochleovestibular signs	18 (94.74)	15 (88.24)	1.99 [0.24–23.5]	0.52		
Tinnitus	11 (57.89)	5 (29.41)	3.07 [0.83–12.5]	0.10		
Dizziness	9 (47.37)	6 (35.29)	1.60 [0.42–5.09]	0.48		
Ataxia	5 (26.32)	2 (11.76)	2.35 [0.48–14.8]	0.30		
Hearing loss, >20 dB	13 (68.42)	12 (70.59)	0.91 [0.22–3.63]	0.90		
Hearing loss, >40 dB	13 (68.42)	8 (47.06)	2.32 [0.63–9.04]	0.20		
Cerebrospinal fluid						
Proteins >0.4 g/L	14 (73.68)	16 (94.12)	0.18 [0.01–2.40]	0.21		
Proteins, g/L	1.1 [0.6–1.8]	1.5 [1.0–2.75]	0.48 [0.18–1.11]	0.10		
MRI^c^						
Number of DWI‐HL	39 [2–85]	89.5 [22.25–200]	0.99 [0.98–1.00]	0.07		
Treatment at diagnosis						
Corticosteroid	16 (84.21)	16 (94.12)	0.60 [0.05–5.01]	0.63		
High‐dose pulse	15 (78.95)	15 (88.24)	0.51 [0.09–2.56]	0.48		
IS drugs	1 (5.26)	6 (35.29)	0.15 [0.01–0.86]	0.03	0.15 [0.01–1.07]	0.06
IVIG	3 (15.79)	8 (47.06)	0.25 [0.05–1.05]	0.06		
Antiplatelet therapy	17 (90)	17 (100)	0.33 [0.01–6.70]	0.48		
Follow‐up						
Duration, months	50.3 [31.4–85.7]	56.1 [22.8–86.6]	1.00 [1.00–1.00]	0.59		
Relapses per patient	1 [0–3]	1 [1–2]	1.01 [0.58–1.77]	0.69		

*Note*: Quantitative variables are expressed as median [1st quartile–3rd quartile]. Qualitative variables are expressed as number (percentage). Severe HL defined as the loss of 70 dB in at least one ear on audiometry or the need for hearing aids at last follow‐up.

Abbreviations: CI, confidence interval; dB, decibel; DWI‐HL, hyperintense lesions on diffusion‐weighted imaging; HL, hearing loss; IS, immunosuppressive; IVIG, intravenous immunoglobulin; MRI, magnetic resonance imaging; OR, odds ratio.

^a^ Univariable analysis.

^b^ Multivariable analysis.

^c^ On first MRI available (*n* = 24) performed during the 3 months following the onset of symptoms in all but one patient.

## DISCUSSION

In 2011, a national clinical‐based cohort (CARESS study) was set up in order to better characterize the epidemiological, clinical, and etiological features of SuS. In the current study, all CARESS patients for which audiometry was performed at time of diagnosis and during follow‐up were available were included (Figure S1). We show that IS treatment given at SuS diagnosis is associated with a lower risk of irreversible cochleovestibular damage.

A diagnostic criteria set has been proposed for SuS [7]. Accordingly, definite SuS is considered in patients with an unequivocal clinical and/or paraclinical involvement of all three main organs (i.e., fulfilling the typical clinical triad). In our study, 34 (94.4%) patients (i.e., 31 at diagnosis and 3 during follow‐up) had the typical triad and thus fulfilled criteria for a definite SuS.

SuS carries a high risk of permanent deafness with more than 50% of patients experiencing severe HL during follow‐up. Our findings confirm that all patients with suspected SuS should have an audiometry even if they have no hearing complaint, as the HL may not be perceived by the patient (around 20% of cases in our series). We did not find any association between brain lesions load assessed by DWI at the acute phase and the risks of cochleovestibular damage. However no patient underwent specific high‐resolution MRI imaging of inner ear structures and VIII nerves [13].

Treatment of SuS is empirical, based on the hypothesis of an autoimmune endotheliopathy supporting the use of IS drugs in addition to corticosteroids. The indication and selection of IS medication are based on experts’ opinions only. In the absence of randomized trials, it remains unclear how much and for how long treatment is required and what is the real impact of IS drugs on the natural history of SuS. Because irreversible cochleovestibular damage appears unpredictable, our findings support a systematic use of IS drugs in patients with SuS.

Our study also has several limitations. First, the conclusions are based on a limited number of patients. The multivariable logistic regression is at risk of overfitting in small samples. Second, only tonal audiometries were performed to assess HL. Third, management of the acute phase of the disease may differ between centers and has changed over time. Fourth, treatment choice was based on disease features and severity at onset, as suggested by a higher proportion of patients with encephalopathy treated with IS drugs (Table S1). In the same line, patients who received IS drugs at diagnosis also received more frequently IVIG (Table S1). Fifth, the small number of patients precluded the analysis of specific IS drugs’ impact on the outcome. Sixth, despite a careful design of the CARESS study, patients included in the cohort study have been excluded from the primary analysis because of missing data, leading to selection bias. However, no difference was observed when comparing the selected patients to those from the whole cohort (Table S2).

Our study has several strengths as CARESS is a prospective study including SuS patients in the acute symptomatic phase. Comprehensive clinical assessment, standardized brain MRI, audiometry, and retinal angiography were systematically performed. The protracted follow‐up helped in analyze the long‐term outcome. An expert committee of internists, ophthalmologists, and neuroradiologists reviewed all cases. To our knowledge, our study is the first to identify risk factors associated with poor auditory outcome in SuS.

In conclusion, the odds of severe HL are lower in patients who received IS drugs at diagnosis.

## AUTHOR CONTRIBUTIONS

**Karim Sacré:** Conceptualization; investigation; funding acquisition; writing – original draft; methodology; validation; writing – review and editing; data curation; supervision. **Marion Peyre:** Investigation; writing – original draft; formal analysis. **Arthur Mageau:** Investigation; formal analysis; writing – review and editing. **Marie‐Cécile Henry Feugeas:** Investigation; writing – review and editing. **Serge Doan:** Investigation; writing – review and editing. **Caroline Halimi:** Investigation; writing – review and editing. **Isabelle Klein:** Conceptualization; investigation; methodology; writing – review and editing. **Tiphaine Goulenok:** Investigation; writing – review and editing. **Chrystelle François:** Investigation; writing – review and editing. **Marie‐Paule Chauveheid:** Investigation; writing – review and editing. **Thomas Papo:** Conceptualization; funding acquisition; supervision; methodology; investigation; writing – review and editing.

## FUNDING INFORMATION

This work was supported by a grant from the French PHRC 2009 (No. P081261) from the Ministère de la Sante, and a grant from the French CRC 2019 (No. CRC19059) from the Ministère de la Santé, the University Paris Cité, and the Assistance Publique Hôpitaux de Paris.

## CONFLICT OF INTEREST STATEMENT

None.

## Supporting information


Data S1

## Data Availability

Anonymized data not published within this article are available from the corresponding author upon reasonable request.

## APPENDIX 1

### AUTHOR: THE ORDER OF NAMES HAS BEEN AMENDED SLIGHTLY AS PRESUMABLY THESE INDIVIDUALS ARE INTENDED TO BE LISTED IN ALPHABETICAL ORDER?

Jean‐Francois Alexandra, Fleur Cohen Aubart, Olivier Aumaitre, Naima Beldjoudi, Marie Bodenant, Jean Capron, Thomas de Brouker, Elisabeth Medeiros de Bustos, Solene De Gaalon, Sabrina Debruxelles, Nicole Delory, Antoine Dossier, Emmanuel Ellie, Fatima Farhi, Sophie Godard‐Ducceschi, Bertrand Godeau, Deborah Grosset‐Janin, Mohamed Hamidou, Cedric Laouenan, Bertand Lapergue, Alain Le Quellec, Anne Catherine Bachoud Levi, Shirine Mohamed, Luc Mouthon, Jean‐Baptiste Noury, Diane Rouzaud, Guillaume Turc, Anne Wacongne.
