# Supplementary material for: Risk factors for severe hearing loss in Susac syndrome: A national cohort study
Source: Eur J Neurol. 2024 Jan 18;31(5):e16211. doi: 10.1111/ene.16211 (PMC11235986; doi:10.1111/ene.16211)
Supplement: Supplementary file 1 — Data S1 [file ENE-31-e16211-s001.docx]

**SUPPLEMENTARY MATERIAL**

**Risk factors associated with severe hearing loss in Susac Syndrome: an observational national cohort study.**

Marion Peyre, Arthur Mageau, Marie-Cécile Henry Feugeas, Serge Doan, Caroline Halimi, Isabelle Klein, Tiphaine Goulenok, Chrystelle François, Marie-Paule Chauveheid, Thomas Papo, Karim Sacré on behalf of the French Susac Study Group

**Table S1 Characteristic of Susac patients who did or did not receive immunosuppressive drugs at diagnosis**

|  | IS drugs at diagnosis,  n=7 | | No IS drugs at diagnosis  n=29 | |
| --- | --- | --- | --- | --- |
| Age, years | 38 [34.5-46.5] | | 32 [25-42] | |
| Diagnostic delay, months | 2 [0.9-2] | | 2 [1-4] | |
| Women, n (%) | 5 (71.4) | | 19 (65.5) | |
| Triad at onset, n (%) | 6 (85.7) | | 25 (86.2) | |
| Neurologic signs, n (%) | 7 (100) | | 29 (100) | |
| Headache | 6 (85.7) | | 22 (75.9) | |
| Encephalopathy | 6 (85.7) | | 13 (44.8) | |
| Behavioral, conduct or mood disorder | 3 (42.9) | | 15 (51.7) | |
| Motor impairment | 1 (14.3) | | 11 (37.9) | |
| Ophtalmic signs, n (%) | 7 (100) | | 26 (89.7) | |
| Visual field loss | 3 (42.9) | | 5 (55.5) | |
| Visual acuity loss | 0 (0) | | 8 (27.6) | |
| Arterial occlusion | 7 (100) | | 23 (79.3) | |
| Hyperfluorescence | 2 (28.6) | | 9 (31.0) | |
| Cochleo-vestibular signs, n (%) | 6 (85.7) | | 27 (93.1) | |
| Tinnitus | 3 (42.9) | | 13 (44.8) | |
| Dizziness | 1 (14.3) | | 14 (48.3) | |
| Ataxia | 0 (0) | | 7 (24.1) | |
| Hearing loss , >20 db | 5 (71.4) | | 20 (69.0) | |
| Hearing loss , >40 db | 5 (71.4) | | 16 (55.2) | |
| CSF |  | |  | |
| Proteins >0.4g/L | 7 (100) | | 23 (79.3) | |
| Proteins, g/L | 1.6 [1.4-2.6] | | 1.12 [1.1-1.7] | |
| MRI at diagnosis* |  | |  | |
| Number of DWI-HL | 116 [36-196] | | 40 [9-127] | |
| Treatment at diagnosis, n (%) |  | |  | |
| Corticosteroid | 7 (100) | | 25 (86.2) | |
| High dose pulse | 7 (100) | | 23 (79.3) | |
| IVIG | 6 (85.7) | | 5 (17.2) | |
| Antiplatelet therapy | 7 (100) | | 27 (93.1) | |
| Follow-up |  |  | |  |
| Duration, months | 63.9 [56.4-88.1] | 50.1 [24-72.4] | |  |
| Relapse per patient, n (%) | 1 [1-2] | 1 [1-2] | |  |

Quantitative variables are expressed as median [1^st^ quartile- 3^rd^ quartile]. Qualitative variables are expressed as a number (percentage); CI, confidence interval; CSF, cerebrospinal fluid; DWI-HL, hyperintense lesions on diffusion-weighted imaging; IS, immunosuppressive; IVIG, intravenous immunoglobulin; MRI, Magnetic resonance imaging.

* on 1st MRI available (n=24) performed during the 3 months following the onset of symptoms in all but one patient.

**Table S2** **Characteristics of selected Susac patients (Patients analyzed) as compared to those from the whole cohort (CarESS Cohort)**

|  | CarESS Cohort,  n=57 | Patients analyzed  n=36 |
| --- | --- | --- |
| Age, years | 34 [25 - 42] | 37 [25 - 43] |
| Diagnostic delay, months | 1.5 [0.95 - 3.25] | 2 [1 - 4] |
| Women | 33 (61.4) | 24 (66.7) |
| Triad at onset | 44 (87.2) | 31 (86.1) |
| Neurologic signs | 55 (96.5) | 36 (100) |
| Headache | 38 (66.6) | 28 (77.8) |
| Encephalopathy | 27 (47.4) | 19 (33.3) |
| Behavioral, conduct or mood disorder | 24 (42.1) | 18 (50) |
| Motor impairment | 15 (26.3) | 12 (33.3) |
| Ophtalmic signs | 50 (87.7) | 33 (91.7) |
| Visual field loss | 14 (24.6) | 8 (22.2) |
| Visual acuity loss | 10 (17.5) | 8 (22.2) |
| Arterial occlusion | 41 (71.9) | 30 (83.3) |
| Hyperfluorescence | 19 (33.3) | 11 (30.5) |
| Cochleo-vestibular signs | 49 (85.6) | 33 (91.7) |
| Tinnitus | 20 (35.1) | 16 (44.4) |
| Dizziness | 22 (38.6) | 15 (41.7) |
| Ataxia | 9 (15.8) | 7 (19.4) |
| CSF |  |  |
| Proteins >0.4g/L | 49 (85.6) | 30 (83.3) |
| Proteins, g/L | 1.16 [0.79 - 1.62] | 1.17 [0.94 - 1.81] |
| MRI at diagnosis* |  |  |
| Number of DWI-HL | 43.5 [13.25 - 127] | 40 [12 - 131] |
| Treatment at diagnosis |  |  |
| Corticosteroid | 52 (91.2) | 32 (88.9) |
| High dose pulse | 45 (78.9) | 30 (83.3) |
| IS drugs | 10 (17.5) | 7 (19.4) |
| IVIG | 16 (28.1) | 11 (30.6) |
| Antiplatelet therapy | 52 (91.2) | 34 (94.4) |
| Follow-up |  |  |
| Duration, months | 51.2 [24.1 - 85] | 51.8 [29.2 -77.6] |
| Relapse per patient | 1 [0-2] | 1 [1 - 2] |

Quantitative variables are expressed as median [1^st^ quartile- 3^rd^ quartile]. Qualitative variables are expressed as a number (percentage); CI, confidence interval; CSF, cerebrospinal fluid; DWI-HL, hyperintense lesions on diffusion-weighted imaging; IS, immunosuppressive; IVIG, intravenous immunoglobulin; MRI, Magnetic resonance imaging.

* on 1st MRI available (n=24) performed during the 3 months following the onset of symptoms in all but one patient.

**Figure S1 Flowchart**

**
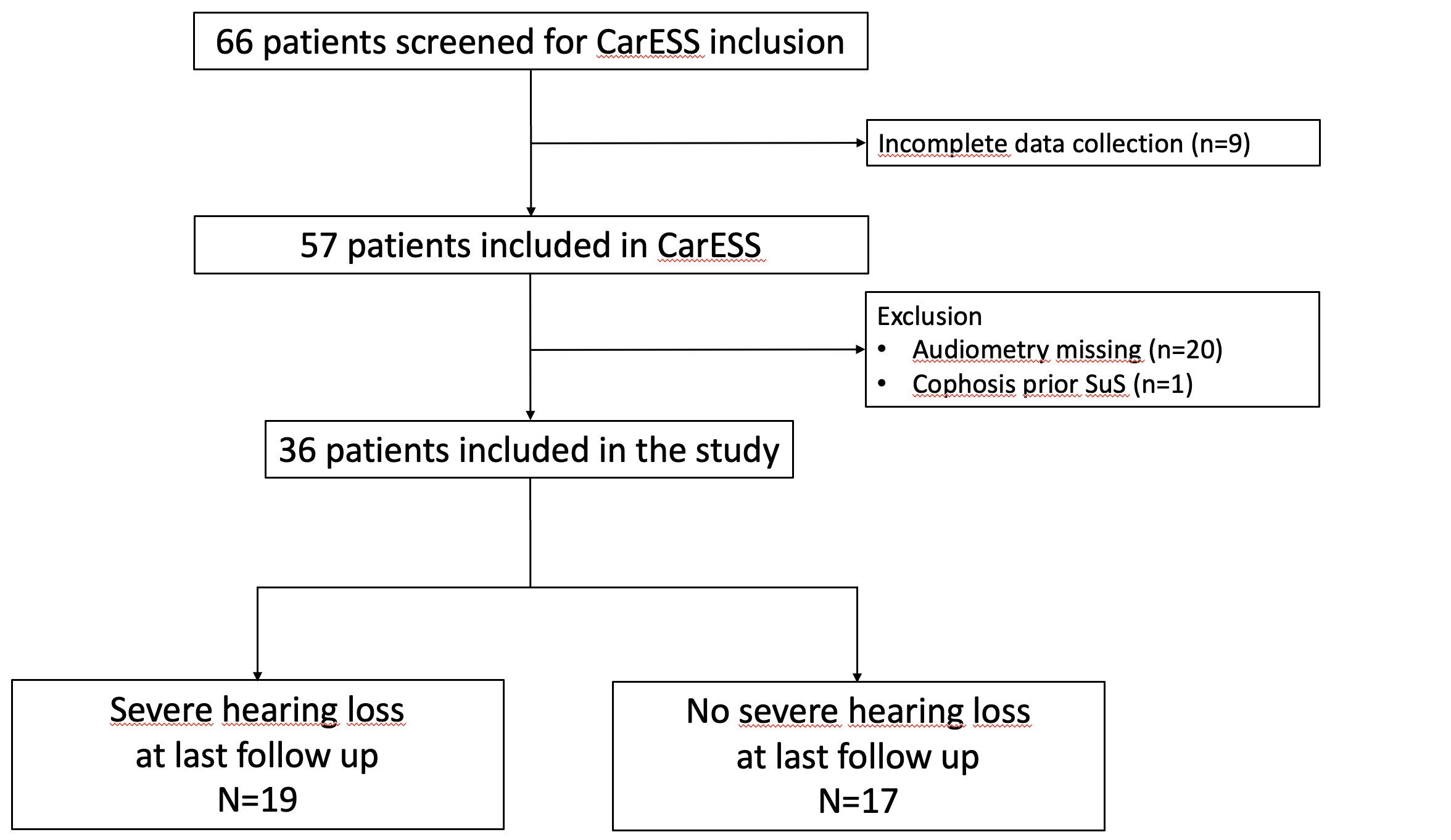
**
